# Cervical Impairments in Subjects with Chronic Migraine: An Observational Study

**DOI:** 10.3390/life13081773

**Published:** 2023-08-18

**Authors:** José Angel del-Blanco-Muñiz, Daniel Martín-Vera, Maria Dolores Sosa-Reina, Alfonso Trinidad-Morales, Marta de-la-Plaza-San-Frutos, Alberto Sánchez-Sierra

**Affiliations:** 1 Faculty of Sport Sciences, Department of Physiotherapy, Universidad Europea de Madrid, 28670 Villaviciosa de Odón, Madrid, Spain; joseangel.delblanco@universidadeuropea.es (J.A.d.-B.-M.); mariadolores.sosa@universidadeuropea.es (M.D.S.-R.); marta.delaplaza@universidadeuropea.es (M.d.-l.-P.-S.-F.); alberto.sanchez@universidadeuropea.es (A.S.-S.); 2 Therapeutic Exercise and Functional Rehabilitation Research Group, Faculty of Sports Sciences, Universidad Europea de Madrid, 28670 Villaviciosa de Odón, Madrid, Spain; 3 Musculoskeletal Pain and Motor Control Research Group, Faculty of Sport Sciences, Universidad Europea de Madrid, 28670 Villaviciosa de Odón, Madrid, Spain; 4 Faculty of Social Sciences and Communication, Department of Education and Humanities, Universidad Europea de Madrid, 28670 Villaviciosa de Odón, Madrid, Spain; alfonso.trinidad@universidadeuropea.es; 5 Aqualab Research Group, Faculty of Social Sciences and Communication, Universidad Europea de Madrid, 28670 Villaviciosa de Odón, Madrid, Spain

**Keywords:** migraine, ultrasound, pain, functionality

## Abstract

Objective: The aim of this investigation was to compare the thickness of the deep local muscles in the neck region, as well as local and widespread sensitivity and functionality, between individuals with migraine and healthy control subjects. Methods: An observational study was carried out in accordance with the STROBE statements. The flexor longus colli and multifidus, two neck-stabilizing muscles, were measured using B-mode ultrasound imaging. The upper trapezius, masseter, temporalis, anterior tibialis, and median nerve all underwent bilateral pressure-pain threshold (PPT) assessments. The statistical program SPSS 29.0 was used to implement the Mann–Whitney U test and Chi-squared test. Spearman Rho was utilized to establish the correlations between the variables. Results: Sixty participants were enrolled in the study. The subjects, who were matched in terms of age, gender, and body mass index (BMI), were equally divided into migraine and control groups. No significant differences between the groups were found in the multifidus CSA regarding both sides at rest (right: *p* = 0.625; left: *p* = 0.203). However, in contraction, the multifidus CSA showed a significant decrease on the left side in the patients with migraine compared to the controls (*p* = 0.032), but no significant differences were found in the right multifidus CSA in contraction between the two groups (*p* = 0.270). In comparison to the healthy volunteers, the migraine sufferers showed a substantial reduction in CSA in the longus colli muscle on both the left side (*p* = 0.001) and the right side at rest (*p* = 0.003), as well as in the CSA of the left longus colli in contraction (*p* < 0.001). Furthermore, the migraine patients showed significantly lower PPT compared to the healthy subjects in local and widespread areas bilaterally. All the parameters revealed higher sensitization in the migraine group in the following areas: the right and left temporal regions (*p* < 0.001), the right and left upper trapezius (*p* < 0.001 and *p* < 0.01, respectively), the right and left masseter muscles (*p* < 0.01), the right and left median nerves (*p* < 0.001 and *p* < 0.01, respectively), and the right and left anterior tibialis muscles (*p* < 0.001)**.** In terms of the craniocervical flexion test (CCFT), the migraine patients demonstrated significantly lower values than the healthy subjects (*p* < 0.001). A moderate positive correlation was noted between the PPT in the right temporalis muscle and that in the left longus colli and the right multifidus in contraction. The PPT in the right temporalis muscle also exhibited a positive correlation with the CCFT, although this correlation was low. Between the PPT values, the upper trapezius on both sides showed a moderate positive correlation with the median nerve bilaterally. Conclusions: This research suggests that individuals with migraine may experience local and widespread pain sensitization. A decrease in functionality due to the low muscle endurance of the deep cervical muscles is also accompanied by low values of muscle thickness in contraction. These findings may help to select more accurate treatment approaches for patients with migraine.

## 1. Introduction

After low back pain, migraine is the pathology that causes the highest rates of disability in the world, ranking first among neurological disorders [1]. Its management generates a high level of economic impact.

One aspect to consider is that data suggest an increase in the prevalence of migraine among the most disabling diseases, as demonstrated by the 2016 study on the Global Burden of Disease [2]. The prevalence of this pathology in developed countries such as the United States is higher in women than in men, with the former ranging around 21% and the latter around 10% [3]. In Spain, the overall prevalence is approximately 12% [4], although there are regional differences, ranging from 7.6% in Navarre to 18% in the Canary Islands. However, these differences in data seem to be due to factors such as altitude, weather, and temperature, or personal factors like alcohol consumption [5].

The International Headache Society (IHS) in its third edition of the International Classification of Headache Disorders [6] describes chronic migraine as a headache that occurs for 15 days or more per month for more than three months, and characteristics of migraine headache present on at least 8 of those days. The clinical diagnostic criteria for chronic migraine are shown in Table 1.

Despite the efforts of the IHS to classify headaches, including chronic migraines, their diagnosis remains challenging as it relies solely on clinical aspects. To date, there are no biological or imaging markers that allow us the classification of headache pain among the numerous subtypes it encompasses.

The classical treatment approach for migraines is pharmacological and has two objectives. Firstly, the administration of preventive medications aims to reduce the frequency of headache episodes. These may include beta blockers, calcium antagonists, neuromodulatory antiepileptics, antidepressants, or angiotensin-converting enzyme inhibitors [7]. On the other hand, there is a group of medications aimed at reducing symptoms during acute episodes, primarily consisting of the administration of non-steroidal anti-inflammatory drugs (NSAIDs) and triptans [8].

However, recent studies assert that the most effective treatment approach in reducing the intensity and frequency of migraine is a multimodal approach that includes manual therapy and exercise [9]. In fact, some international scientific societies, such as the French Headache Society, include physical exercise as part of the non-pharmacological treatment for migraine [10].

Just as in tension-type headache (TTH), there is no doubt about its association with musculoskeletal dysfunctions, such as reduced cervical range of motion and forward head posture [11] or the presence of trigger points in the cranio-cervical region [12]. However, the evidence is not sufficiently robust due to the limited number of observational studies concerning migraine. Some existing studies have shown a decrease in cervical range of motion in migraine patients when compared with healthy individuals [13]. Additionally, reduced pain levels upon pressure in the frontal, temporal, and upper trapezius musculature were noted [14] along with a decreased strength in the cervical extensor and flexor muscles [15].

The recommendation for applying manual therapy in patients with migraine is supported by the higher prevalence of cervical pain in these patients compared to the general population. In certain studies [16], a statistically significant difference in the presence of cervical pain was observed between patients without migraine, estimated at 56.7%, compared to subjects with any type of primary headache, with a prevalence of 89.3% in subjects with migraine + TTH, 88.4% in subjects with pure TTH, and 76.2% in migraine sufferers. Luedtke [13] analyzed the effect of oscillatory joint mobilization and sustained pressure applied by a blinded physiotherapist on upper cervical joint structures in 179 subjects diagnosed with migraine and 73 healthy subjects. In 47% of migraine subjects, the referred pain was reproduced towards the head, recognized by the subjects as their headache pain.

These findings could be attributed to the convergence of sensory afferents from both the trigeminal nerve and upper cervical nerves in the trigeminal cervical nucleus [17]. According to Luedtke and May [13], these musculoskeletal dysfunctions at the cervical level should be treated with manual therapy to prevent ongoing nociceptive input into the trigeminal–cervical nucleus.

Based on the prevalence of cervical musculoskeletal dysfunctions in patients with migraine, another aspect that reinforces the relationship between migraine symptoms and cervical dysfunctions is the results of different studies demonstrating improvement in headache-related symptoms when manual therapy is applied to that anatomical region. For example, in the study by Luedtke [18], manual therapy focused on the cervical region led a decreased pain intensity (MD −1.94, −2.61 to −1.27) and migraine duration (MD −22.4 h, −33.9 to −10.9).

In another experimental study [19], a total of 50 patients with migraine were randomly assigned into two groups: an intervention group and a placebo group. The migraine group received four sessions of articulatory manual therapy applied to the cervical region. After the completion of these four sessions, the results indicated a significant reduction in pain, improvement in quality of life, and decreased disability and medication usage within the manual therapy group compared to the placebo group.

However, in a recent systematic review, it was concluded that the methodological quality of studies focusing on migraine and cervical dysfunctions remains low, emphasizing the need for further research with higher methodological rigor [20]. 

Consequently, the objective of this study is to elucidate distinct and objectively measurable exploratory findings in patients with migraine as compared to healthy subjects. These findings may help practitioners in determining the most appropriate non-pharmacological treatment strategies.

## 2. Methods

### 2.1. Study Design

An observational study was conducted following the guidelines outlined by the Reporting of Observational Studies in Epidemiology (STROBE) Initiative Statement [21]. The study adhered to the principles set forth in the 1964 Declaration of Helsinki and received approval from the Research Ethics Committee of the Rey Juan Carlos University of Madrid (Spain).

### 2.2. Participants

Participant recruitment took place between September and November 2022 at university facilities in the region of Madrid, Spain. The investigation included sixty participants, divided evenly into two groups: a migraine group and a control group, without migraine. A comparison between subjects with migraine and control subjects was performed. Individuals in the migraine group had previously received a diagnosis of chronic migraine from a neurology specialist based on the criteria outlined in the third edition of the International Classification of Headache Disorders (ICHD-3) (ICHD-3 Beta). Eligible participants met the following criteria: (1) Adults aged 18 to 65 years; (2) Suffering from chronic migraine for a duration exceeding six months. Exclusion criteria encompassed: (1) Presence of any nervous system disorder. (2) Pregnancy. (3) Botulinum toxin injection in the last 2 months. Volunteers in the control group had not experienced chronic painful disorders in the preceding 6 months.

### 2.3. Procedure

Prior to enrollment, patients were provided with an information sheet detailing all investigative procedures and were required to provide informed consent upon comprehension. Patients also received verbal information and had the opportunity to address any queries with researchers. An experienced examiner, well-versed in the protocol, familiarized themselves with the procedure before the first patient was enrolled. The protocol´s integrity was verified by two different members of the research team to mitigate potential bias.

### 2.4. Outcomes

Anthropometric variables were recorded using Shephard’s methodology, with specific attention given to measurements of height, weight, and body mass index (BMI) [22]. Height and weight data were collected using a tape measure and a numerical scale, respectively.

### 2.5. Characteristics of Migraine Episodes

Descriptions regarding the key features of migraine episodes are crucial for comprehending the daily suffering experienced by patients. Among these, intensity (measured on a scale from 0 to 10), duration (in hours) and frequency (in days), were considered as the primary variables, following the methodology of a previous study [23].

### 2.6. Muscle Thickness

**Figure 1:**
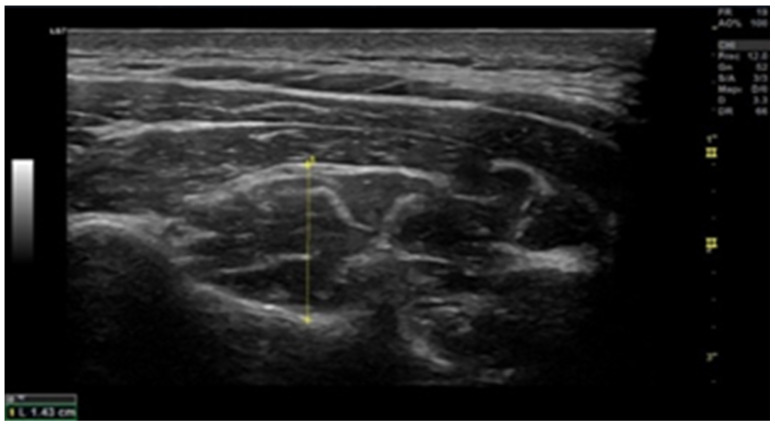
Multifidus ultrasound image at C5 (transverse view). The probe is placed 90° to the lamina of C5 where the rater considered the muscle to be at its thickest and up to the echogenic line of the hyperechoic fascia between the semispinalis cervicis and semispinalis capitis.

**Figure 2:**
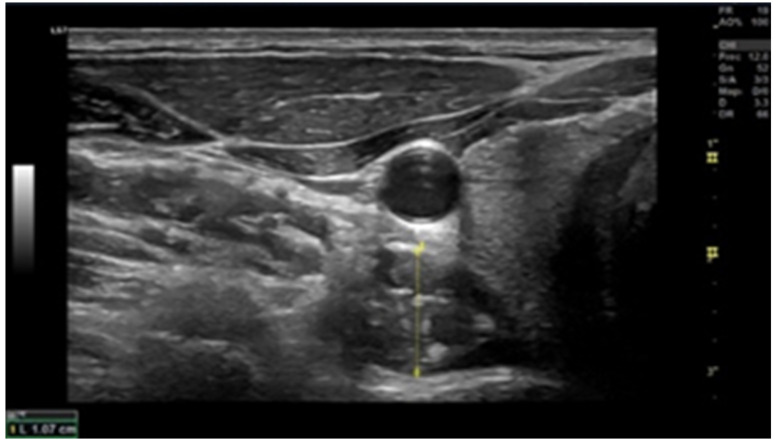
Longus colli ultrasound image at C6 (transverse view). The probe is placed on the midpoint of the ventral surface of the C6 vertebral body and the interface between the Lcol and the pre-fascial tissue surrounding the carotid artery.

Ultrasound scans were conducted using a high-resolution device (GE Healthcare, Chicago, USA) to assess the cross-sectional area (CSA). The examination of deep local muscles was performed at the C5–C6 level (Figure 1 and Figure 2). Ultrasound measurements were recorded bilaterally, both in a resting position and during the contraction of multifidus and flexor longus colli muscles [24]. To ensure muscle relaxation prior to obtaining the resting measurement, patients were instructed to remain in a relaxed state, while the examiner used palpation to monitor any sign of muscle tension. For the contraction measurement, participants executed either a “double chin” maneuver (bringing the chin to the sternum) or neck extension, depending on the muscle measured. This procedure was initially demonstrated by the researcher, aiding patients in becoming familiar with the process (Øverås et al., 2017).

### 2.7. Pressure Pain Threshold (PPT)

The protocol established by Fleckenstein et al. [25] was replicated in this study. A FORCE DIAL FDK/FDN 100 algometer (Wagner Instruments, Greenwich, CT, USA) was employed as an optimal measuring device. Presently, PPT has demonstrated the highest reliability as a mechanical threshold recording method [26]. Participants were assessed in a relaxed supine position. Bilateral PPT measurements were taken in masseter, temporalis, upper trapezius, anterior tibialis and median nerve. The precise spots were as follows:

- Trapezius: Midway between C7 and the acromion.
- Masseter: 1 cm above the insertion point of the superficial portion at the angle of the jaw.
- Temporalis: The middle fibers in the bone depression approximately 2 cm lateral to the outer edge of the eyebrow.
- Anterior tibialis: upper one third of the muscle belly.
- Median nerve: in the flexion crease of the elbow, located from the biceps brachii tendon at the elbow.

Exact points were marked to reproducibility, and measurements were taken 3 times. The algometer was applied to each point, gradually increasing pressure at the rate of approximately 1 kg/cm^2^ per second.

Patients were familiarized with the procedure prior to recording any PPT measurement. Subjects were required to notify the examiner when they first felt a painful sensation upon pressure from the rubber tip of the algometry device. PPT measurements using the algometer exhibited good to excellent interrater reliability (ICC: 0.64–0.92) and test–retest reliability (ICC: 0.72–0.95).

### 2.8. Craniocervical Flexion Test (CCFT)

CCFT was employed to assess the functionality of local muscles in the cervical region using the Stabilizer Pressure biofeedback device (Chattanooga Group, Hixon, TN, USA), following the methodology outlined by Thongprasert and Kanlayanaphotporn [27]. Subjects assumed a supine position with semi-flexed knees, maintaining a neutral neck position. The Stabilizer was positioned in the suboccipital area and inflated to an initial pressure of 20 mmHg. Subjects were instructed to perform craniocervical flexion by nodding while the pressure increased incrementally by 2 mmHg from 20 to 30 mmHg. Each increment was held for 10 s. This approach allowed the assessment by the examiner of the subject´s ability to sustain the contraction without displaying signs of fatigue, thus measuring the endurance capacity of anterior deep cervical muscles (flexor longus colli). 

Fatigue signs were monitored by palpating the superficial neck flexor muscles. The activation score (AS) and the performance index (PI) were derived from CCFT. The AS represented the maximum pressure level that the subjects could consistently tolerate for 10 seconds. The PI, focused on assessing the endurance capacity of the deep cervical flexor muscles, was calculated based on the number of times the test could be repeated at the AS. The maximum PI score of 100 (10 repetitions at 10 mmHg AS) was established. Data were transformed into a rating based on other studies, with an anomalous response considered as ≤4 mmHg for AS and ≤20 scores for Pl. The interrater reliability ICC score was 0.89, and the 95% confidence interval was between 0.70 and 0.94. The intra-rater reliability ICC score was 0.87, with a 95% confidence interval being between 0.77 and 0.93. Consequently, the Stabilizer device exhibited good to exceptional inter- and intra-rater reliability [28].

### 2.9. Data Collection Methods

Data were collected through self-reported forms and physical examination conducted by a single researcher. The datasets employed and analyzed in this study can be made available by the corresponding author upon reasonable request.

### 2.10. Statistical Analysis

Statistical analysis was performed using the SPSS program (version 29.0) and graphic representation was accomplished using the Sigma Plot program (version 11.0). The normality of variable distribution was assessed using the Shapiro–Wilk test. Quantitative variables were presented as median and interquartile range, while data from qualitative variables were reported as absolute and relative frequencies. Group comparisons were conducted using the non-parametric Mann–Whitney U test for quantitative variables and the Chi-square test for qualitative variables. Correlations were determined using Spearman’s Rho test.

## 3. Results

### 3.1. Characteristics of the Enrolled Participants

A total of 60 participants (30 migraine patients and 30 healthy individuals) ultimately took part in the study. Anthropometric measurements were gathered from both groups (Table 2). The findings demonstrate that there were no statistically significant differences in sex, age, weight, and height (*p* > 0.05) between healthy subjects and migraine patients.

### 3.2. Headache Characteristics

The headaches exhibited an average frequency of 11.00 ± 8.78 days per month, an average intensity of 7.22 ± 1.33, and an average duration of 10.91 ± 8.23 h (Table 3).

### 3.3. Muscle Thickness

No significant differences were observed in the left multifidus CSA between healthy subjects and migraine patients (1.26 [1.34–1.17] vs. 1.23 [1.29–1.13]; *p* = 0.203). Similarly, there were no statistically significant differences in the right multifidus CSA between the two groups at rest (1.24 [1.36–1.15] vs. 1.24 [1.30–1.16]; *p* = 0.625).

However, in terms of the left multifidus CSA during contraction, a significant difference was found in the left-side CSA of the contracted multifidus (1.42 [1.58–1.32] vs. 1.37 [1.41–1.29]; *p* = 0.032), indicating a reduction in migraine patients compared to healthy subjects. Conversely, differences in the right multifidus CSA during contraction between the two groups did not achieve statistical significance (1.40 [1.54–1.31] vs. 1.38 [1.43–1.31]; *p* = 0.270). 

**Figure 3:**
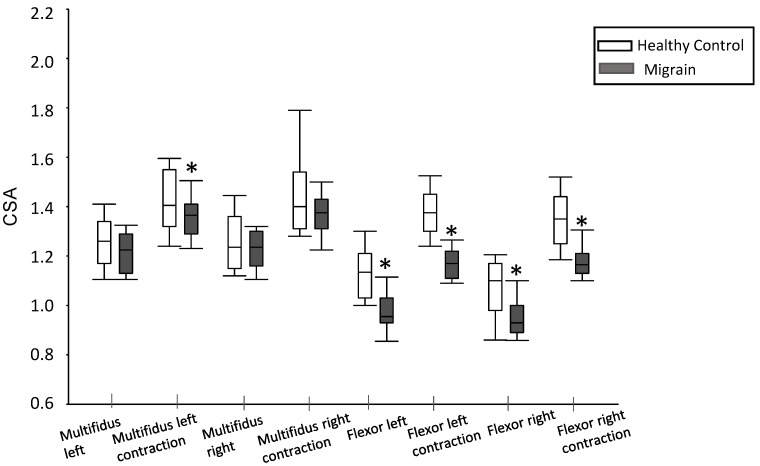
Results of cross-sectional area (CSA) in migraine patients compared to healthy subjects. *: *p* < 0.05 between the two groups.

Furthermore, concerning to the longus colli muscle, a significant reduction in cross-sectional area was observed both on the left side (0.96 [1.03–0.93] vs. 1.14 [1.21–1.03]; *p* < 0.001) and the right side (0.93 [1.00–0.89] vs. 1.10 [1.17–0.98]; *p* = 0.003) in migraine patients compared to healthy subjects at rest. Additionally, a significant decrease in the CSA of the left longus colli muscle during contraction was also evident in migraine patients compared to healthy subjects (1.17 [1.21–1.11] vs. 1.35 [1.44–1.25]; *p* < 0.001) (Figure 3).

### 3.4. Pain Pressure Threshold

**Figure 4:**
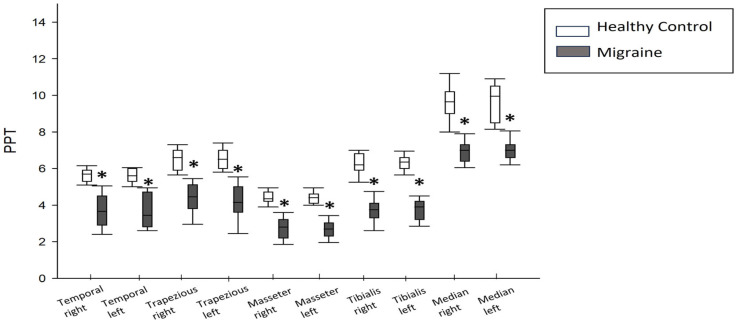
Results of pain pressure threshold (PPT) in migraine patients compared to healthy subjects. *: *p* < 0.05 between the two groups.

Numerous parameters were subjected to comparison between the two groups of participants. Migraine patients exhibited significantly lower pain pressure thresholds in comparison to healthy subjects across various regions, including the right and left temporal regions (3.65 [4.50–2.90] vs. 5.70 [5.90–5.30]; *p* < 0.001 and 3.50 [4.7–2.8] vs. 5.60 [6.0–5.3]; *p* < 0.001, respectively), right and left upper trapezius (4.50 [5.1–3.8] vs. 6.6 [7.0–5.9]; *p* < 0.001 and 4.15 [5.0–3.6] vs. 6.50 [7.00–6.00]; *p* < 0.01, respectively), right and left masseter muscles (2.80 [3.20–2.20] vs. 4.35 [4.70–4.20]; *p* < 0.01 and 2.69 [3.03–2.30] vs. 4.40 [4.60–4.10]; *p* < 0.01, respectively), right and left median nerves (3.8 [4.1–3.3] vs. 6.2 [6.8–5.9]; *p* < 0.001 and 3.9 [4.2–3.2] vs. 6.4 [6.60–6.00]; *p* < 0.01, respectively), and right and left anterior tibialis muscles (7.00 [7.3–6.4] vs. 9.7 [10.2–9.00]; *p* <0.001 and 7.00 [7.3–6.4] vs. 10 [10.5–8.5]; *p* < 0.001, respectively) (Figure 4).

### 3.5. Craniocervical Flexion

In relation to craniocervical flexion, it was observed that migraine patients obtained significantly lower values in the test compared to healthy subjects (20 [24–22] vs. 24 [24–22]; *p* < 0.001).

### 3.6. Correlations

The obtained results revealed several correlations among the analyzed variables in the study. A notably strong positive correlation was evident between the right temporal pain pressure threshold (PPT) and the left temporal PPT (rho = 0.780; *p* < 0.001), along with a minor negative correlation with the left tibial PPT (rho = −0.383; *p* = 0.037) and a moderately strong positive correlation with the contracted CSA of the right-sided multifidus and left-sided contracted longus colli CSA (rho = 0.447; *p* = 0.013 and rho = 0.427; *p* = 0.019, respectively). Additionally, a lower positive correlation, although statistically significant, was observed between the left temporal PPT and the left tibial PPT and the craniocervical flexion (rho = 0.395; *p* = 0.031 and rho = 0.371; *p* = 0.043, respectively). Furthermore, it also exhibited a moderately strong positive correlation with the relaxed and contracted CSA of the right-sided multifidus (rho = 0.447; *p* = 0.013 and rho = 0.455; *p* = 0.012, respectively).

The right upper trapezius PPT showed a high positive correlation with the left upper trapezius PPT (rho = 0.773; *p* < 0.001) and a moderate positive correlation with the right and left median nerve PPT (rho = 0.458; *p* = 0.011 and rho = 0.467; *p* = 0.009, respectively). The right upper trapezius PPT also correlated with the left median nerve PPT (rho = 0.384; *p* = 0.036).

A very high strong positive correlation was evident between the PPT of the right and left masseter (rho = 0.852; *p* < 0.001) and a moderately strong negative correlation with the contracted CSA left-sided longus colli (rho = −0.499; *p* = 0.005) and the right-sided contracted longus colli CSA (rho= −0.439; *p* = 0.015). The PPT of the left masseter exhibited a moderate positive correlation with the PPT of the left median nerve (rho = −0.427; *p* = 0.019).

The PPT of the right median nerve showed a very strong positive correlation with the PPT of the left median nerve (rho = 0.925; *p* < 0.001). Additionally, the PPT of the right tibialis anterior demonstrated a notably strong positive correlation with the PPT of the left tibialis anterior (rho = 0.859; *p* < 0.001).

The CSA of the left-sided multifidus correlated very strongly with the contracted multifidus CSA (rho = 0.889; *p* < 0.001) and positively and moderately with the right-sided relaxed and contracted CSA of the multifidus (rho = 0.575; *p* < 0.001 and rho = 0.527; *p* = 0.003, respectively). Furthermore, the contracted CSA of the left-sided multifidus correlated moderately and positively with the relaxed and contracted CSA of the right-sided multifidus (rho = 0.414; *p* = 0.023 and rho = 0.534; *p* = 0.002, respectively). The CSA of the right-sided multifidus, in turn, correlated with the contracted multifidus CSA (rho = 0.723; *p* < 0.001), and the latter exhibited a weak positive correlation with the contracted CSA of the left-sided longus colli (rho = 0.363; *p* < 0.048). The contracted CSA of the left-sided longus colli correlated highly positively with the contracted longus colli CSA and with the relaxed and contracted CSA of the right-sided longus colli (rho = 0.628; *p* < 0.001, rho = 0.648; *p* < 0.001, rho = 0.688; *p* < 0.001, respectively). Furthermore, the contracted CSA of the left-sided longus colli demonstrated moderate to high positive correlations with the relaxed and contracted CSA of the right-sided longus colli (rho = 0.589; *p* > 0.001 and rho = 0.779; *p* < 0.001, respectively). Lastly, a strong positive correlation was observed between the CSA of the longus colli and the contracted CSA of the longus colli (rho = 0.651; *p* < 0.001).

## 4. Discussion

The objective of this investigation was to compare the functional and anatomical characteristics of individuals with migraine to those of healthy individuals.

In this study, a decrease in strength during the craniocervical flexion test among migraine patients was observed. These findings are consistent with those of previous studies that have demonstrated alterations in the deep neck muscles, including decreased CSA and strength [15].

Notably, trigger point presence and decreased pain pressure threshold in the frontal, temporal, and upper trapezius muscles, as previously documented [12,14], were also observed in our study.

Concerning muscle thickness, an intriguing observation emerged when analyzing the data for the extensor muscles (multifidus). A significant change in CSA was observed only in the left multifidus of migraine patients compared to healthy individuals. This finding aligns with those of Almazán-Polo et al. [29], who also demonstrated this asymmetry between the left and right multifidus in patients with chronic low back pain. Similarly, Hides [30] reported that a 1 cm^2^ asymmetry in multifidus at the L5 level predicted lower limb injuries with a 92% accuracy. Although we are investigating different anatomical regions (cervical and lumbar), the function of the multifidus muscles is similar, and the asymmetry between the right and left sides may contribute to exacerbating issues in migraine patients.

While strength training could be an effective recommendation for alleviating migraine symptoms [31], migraine patients might feel uncomfortable while exercising due to the random and occasionally intense onset of migraine episodes. As a result, this discomfort may lead to lower adherence to different exercise programs and potential atrophy of deep cervical muscles, as found in this study.

Furthermore, this study confirms that individuals with migraine exhibit alterations in flexor muscles, not only in terms of thickness (as seen in ultrasound measurements at rest and during contraction) but also in terms of strength [15].

Results from the craniocervical flexion test indicate a significant strength decrease (*p* < 0.001). It is reasonable to infer that decreased longus colli muscle thickness leads to diminished flexion strength [15] and head positioning (Liang et al., 2019). Moreover, as Anarte-Lazo et al. previously demonstrated, the CCFT proves valuable for evaluating migraine patients, distinguishing them from cervicogenic headache sufferers and healthy individuals. This test reveals that migraine patients exhibit lower values compared to both groups [20]. This trend is shared with other types of primary headache, like TTH. Previous studies confirm lower values of CCFT in TTH patients compared to healthy individuals, as shown in a recent meta-analysis [11].

In our study, PPT measurements at all assessed points (masseter, temporalis, upper trapezius, anterior tibialis, and median nerve) demonstrated increased sensitization in migraine patients compared to the control group. The strong correlation between the right and left sides, as well as the results obtained for the anterior tibialis muscle and median nerve, suggest that these patients manifest central sensitization. This supports the notion that migraine sufferers have impaired conditioned pain modulation, potentially contributing to migraine headache development. This impairment in the nociceptive inhibitory systems has been stablished in previous studies through the analysis of the nociceptive blink reflex [32].

## 5. Conclusions

The present investigation confirms a higher prevalence of musculoskeletal alterations in patients with chronic migraine compared to healthy subjects. Among all, a diminution of the flexor endurance capacity from the anterior deep cervical muscles has been found. This finding also occurs in other primary headaches, such as TTH. Atrophy changes have been detected in of both posterior and anterior deep cervical muscles, indicating potential objective anatomical variations. Diminished PPT values correlate with higher pain sensitization levels, both local and extensively. Consequently, the endogenous descending inhibitory pain modulation systems may be compromised in patients with chronic migraine, as occurs in other chronic painful conditions.

## 6. Clinical Implications

The study’s findings of low muscle strength and endurance capacity in deep cervical muscles of chronic migraine patients, along with decreased muscle thickness and altered pain sensitization, suggest that investigating muscle-targeted treatments, such as tailored manual therapy or cervical region strengthening programs, could be beneficial. These approaches are already employed for patients with TTH and cervicogenic headache.

## Figures and Tables

**Table 1:** Diagnostic criteria for chronic migraine according to the ICHD-3.

A	Headache (tension-type or migraine-like) occurring for a period of ≥15 days/month for >3 months, fulfilling criteria B and C.
B	It occurs in patients who have experienced at least five attacks fulfilling criteria B-D for 1.1, migraine without aura and/or criteria B and C for 1.2, migraine with aura
C	During a period of ≥8 days/month for >3 months, fulfilling any of the following: Criteria C and D for 1.1, migraine without aura. Criteria B and C for 1.2, migraine with aura. At the time of onset, the patient believes it is a migraine and is relieved by a triptan or ergot derivative.
D	Not attributable to another diagnosis in the ICHD-III.

**Table 2:** Characteristics of the participants.

	Healthy Controls (n = 30)		Migraine Patients (n = 30)		*p* Value
Sex n (%)	Men	13 (43.3%)	Men	10 (33.3%)	0.426
	Women	17 (56.7%)	Women	20 (66.7%)	
Age (years)	29 [46–23]		28 [43–23]		0.90
Weight (Kg)	68 [74–57]		63 [71–61]		0.66
Height (m)	1.67 [1.75–1.60]		1.65 [1.74–1.61]		0.71

Data are presented as absolute or relative frequency or as median and interquartile range (75th percentile–25th percentile). Kg: Kilograms; m: meters.

**Table 3:** Headache characteristics.

	Mean (SD)
Frequency (days in four weeks)	11.00 (8.78)
Intensity (VAS 0–10)	7.22 (1.33)
Duration (h/day)	10.91 (8.23)

VAS: visual analogue scale; SD: standard deviation.

## Data Availability

Data will be available under request to the corresponding author: daniel.martin2@universidadeuropea.es.
